# From one to all: self-assembled theranostic nanoparticles for tumor-targeted imaging and programmed photoactive therapy

**DOI:** 10.1186/s12951-019-0450-x

**Published:** 2019-02-02

**Authors:** Xianlei Li, Xuan Wang, Caiyan Zhao, Leihou Shao, Jianqing Lu, Yujia Tong, Long Chen, Xinyue Cui, Huiling Sun, Junxing Liu, Mingjun Li, Xiongwei Deng, Yan Wu

**Affiliations:** 1 CAS Key Laboratory for Biomedical Effects of Nanomaterials and Nanosafety, CAS Center for Excellence in Nanoscience, National Center for Nanoscience and Technology, No. 11 First North Road, Zhongguancun, Beijing, 100190 China; 2 University of Chinese Academy of Sciences, Beijing, 100049 People’s Republic of China; 3 The First Affiliated Hospital of Jiamusi University, Jiamusi, 154003 China

**Keywords:** From one to all, Self-assembling, Theranostic nanoparticles, Targeted imaging, Programmed photoactive therapy

## Abstract

**Background:**

In recent years, multifunctional theranostic nanoparticles have been fabricated by integrating imaging and therapeutic moieties into one single nano-formulations. However, Complexity of production and safety issues limits their further application.

**Results:**

Herein, we demonstrated self-assembled nanoparticles with single structure as a “from one to all” theranostic platform for tumor-targeted dual-modal imaging and programmed photoactive therapy (PPAT). The nanoparticles were successfully developed through self-assembling of hyaluronic acid (HA)-cystamine-cholesterol (HSC) conjugate, in which IR780 was simultaneously incorporated (HSCI NPs). Due to the proper hydrodynamic size and intrinsic targeting ability of HA, the HSCI NPs could accumulate at the tumor site effectively after systemic administration. In the presence of incorporated IR780, in vivo biodistribution and accumulation behaviors of HSCI NPs could be monitored by photoacoustic imaging. After cellular uptake, the HSCI NPs would disintegrate resulting from cystamine reacting with over-expressed GSH. The released IR780 would induce fluorescence “turn-on” conversion, which could be used to image tumor sites effectively. Upon treatment with 808 nm laser irradiation, PPAT could be achieved in which generated reactive oxygen species (ROS) would produce photodynamic therapy (PDT), and subsequently the raised temperature would be beneficial to tumor photothermal therapy (PTT).

**Conclusion:**

The self-assembled HSCI NPs could act as “from one to all” theranostic platform for high treatment efficiency via PPAT pattern, which could also real-time monitor NPs accumulation by targeted and dual-modal imaging in a non-invasive way.
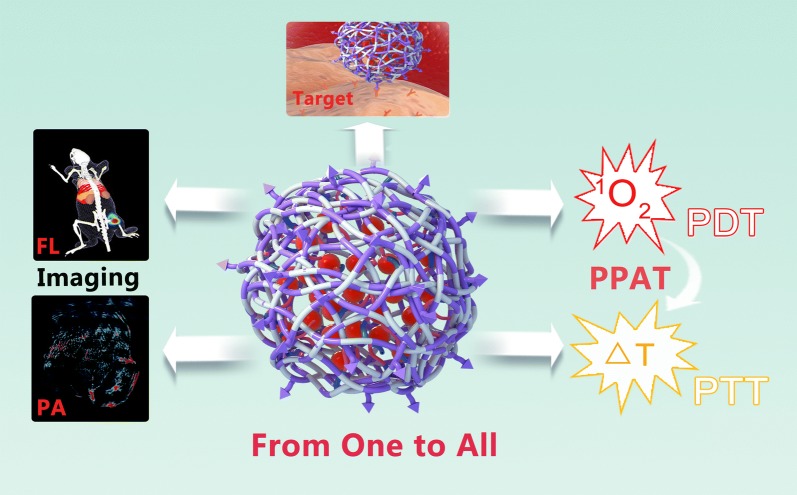

**Electronic supplementary material:**

The online version of this article (10.1186/s12951-019-0450-x) contains supplementary material, which is available to authorized users.

## Background

Blossom of smart and multifunctional theranostic nanoparticles [1–9], paralleled by advances in nanotechnology and other interdisciplinary sciences, is gradually provoking precision medicine into reality [10–14]. The theranostic nanoparticles integrating targeting, imaging and therapeutic abilities into one single nano-formulation could monitor drug accumulation in a real-time manner, allow precise disease diagnosis and evaluate treatment efficiency [15, 16]. However, in consideration of safety factors, complexity of production process and less than idea in vivo pharmacokinetics, most of theranostic nanomedicine is still at academic stage without further application in clinical translation [17–19]. Therefore, exploring theranostic nanoparticles constructed with safe materials and simple preparation method would be beneficial to further application.

Enormous molecular imaging strategies, including magnetic resonance imaging (MRI), positron emission tomography (PET), CT and fluorescence imaging have been widely applied in cancer theranostics [20–23]. By virtue of the outstanding sensitivity, low cost and short acquisition time, fluorescence imaging is broadly employed in (pre)clinical research for disease diagnosis and monitoring the in vivo behaviors of nanoparticles. Nevertheless, poor spatial resolution of fluorescence hinders its further application [24, 25]. As a hybrid imaging modality, multispectral optoacoustic tomography (MSOT) could conquer the optical diffusion limitation via combining the spectral selectivity of molecular excitation with the high resolution of ultrasound detection, which is based on the photoacoustic (PA) effect [26, 27]. On the one hand, outstanding sensitivity of fluorescence could be used to image and track the in vivo behaviors of nanoparticles. On the other hand, high spatial resolution of MSOT could be used to noninvasively monitor drug accumulation behaviors in vivo. Therefore, integrating fluorescence and MSOT imaging into one single nanoplatform represent effective means to boosting its further application and development [28, 29].

Light-triggered photoactive therapy utilizing photo-conversion of photosensitizer could produce reactive oxygen species (ROS) via singlet-to-triplet for photodynamic therapy (PDT) [30, 31] or high temperature for photothermal therapy (PTT) [32–34]. Photoactive therapy has many advantages including noninvasiveness, selective local treatment, negligible drug resistance and minimized side effects. More importantly, incorporating PDT and PTT together can achieve more effective therapeutic effects [35, 36]. Therefore, realizing programed photoactive therapy (PPAT) together with PDT and PTT on one domain would be a superior strategy for cancer treatment. However, combining multiple moieties with different functions would bring about more uncertainties in drug absorption, distribution, metabolism, excretion and toxicity. Therefore, realizing multiple functions in one single platform would be crucial.

**Scheme 1:**
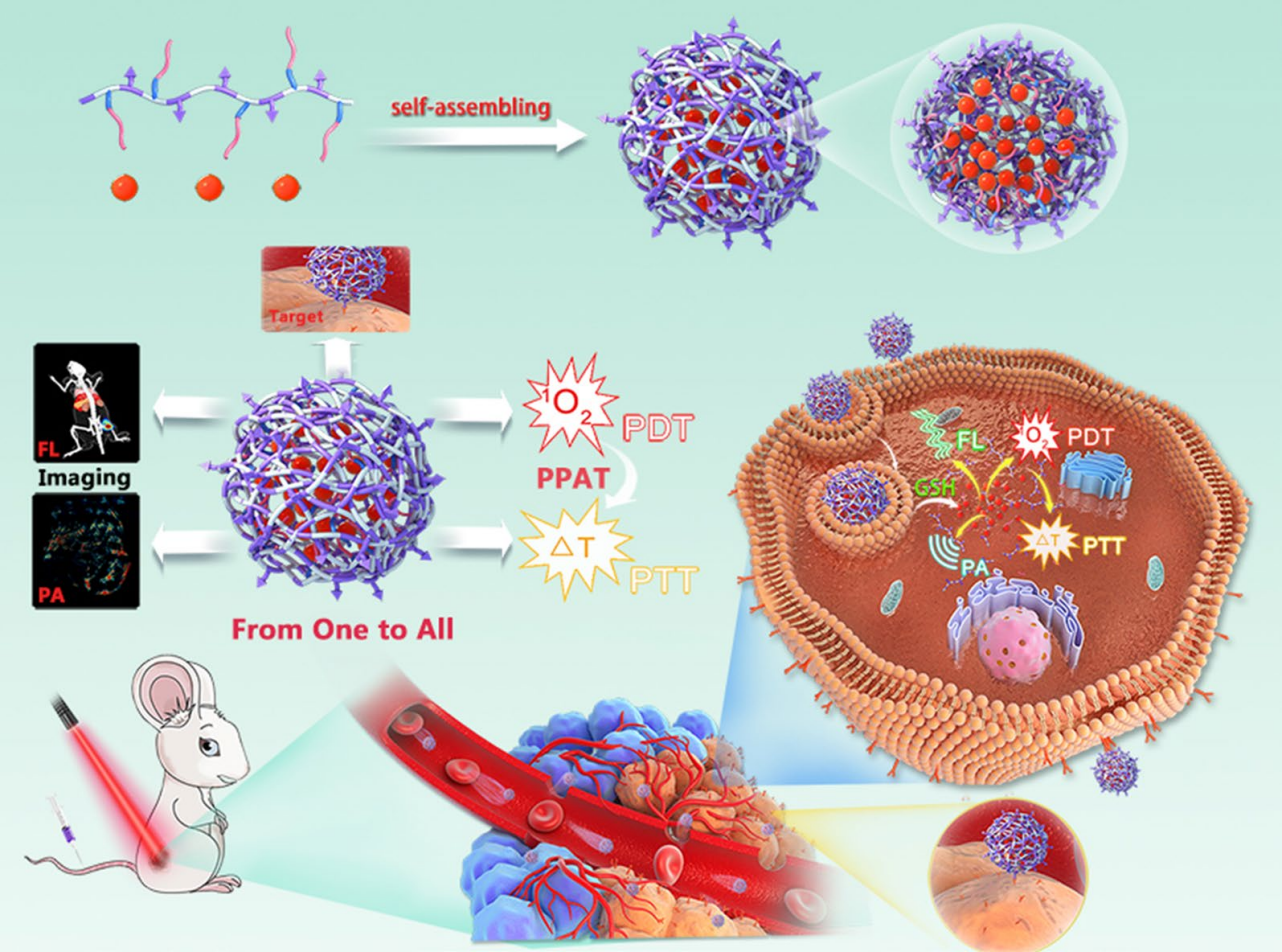
Schematic illustration of the design, preparation and application of “From One to All” theranostic HSCI NPs with multimodal tumor-targeted imaging and programed photoactive therapy (PPAT) in vivo

In the present study, we demonstrated HSCI NPs as “from one to all” theranostic platform for tumor-targeted dual-modal imaging and programmed photoactive therapy (PPAT; PDT followed by PTT). As shown in Scheme 1, the self-assembled HSCI NPs could realize specific accumulation at tumor site benefiting from proper hydrodynamic size and targeting ability of hyaluronic acid (HA) for triple negative breast cancer cells over-expressing CD44 receptor. Attributing to intrinsic imaging property of IR780, the accumulation behaviors of HSCI NPs in vivo could be monitored and tracked by MSOT. Subsequently, the HSCI NPs would disintegrate resulting from cystamine reacting with overexpressed intracellular GSH. The released IR780 would induce fluorescence “turn-on” conversion, which could be used to image tumor sites effectively. Furthermore, NIR laser of 808 nm was implemented for PPAT where PDT functioned at starting and PTT could be realized as the second step. In addition, the HSCI NPs had excellent biosafety and biocompatibility. All together, we developed “from one to all” theranostic nanoparticles constructed with simple self-assembling method, which could realize valid tumor-targeted dual-modal imaging and programmed photoactive therapy.

## Results and discussion

### Materials preparation and characterization

**Fig. 1:**
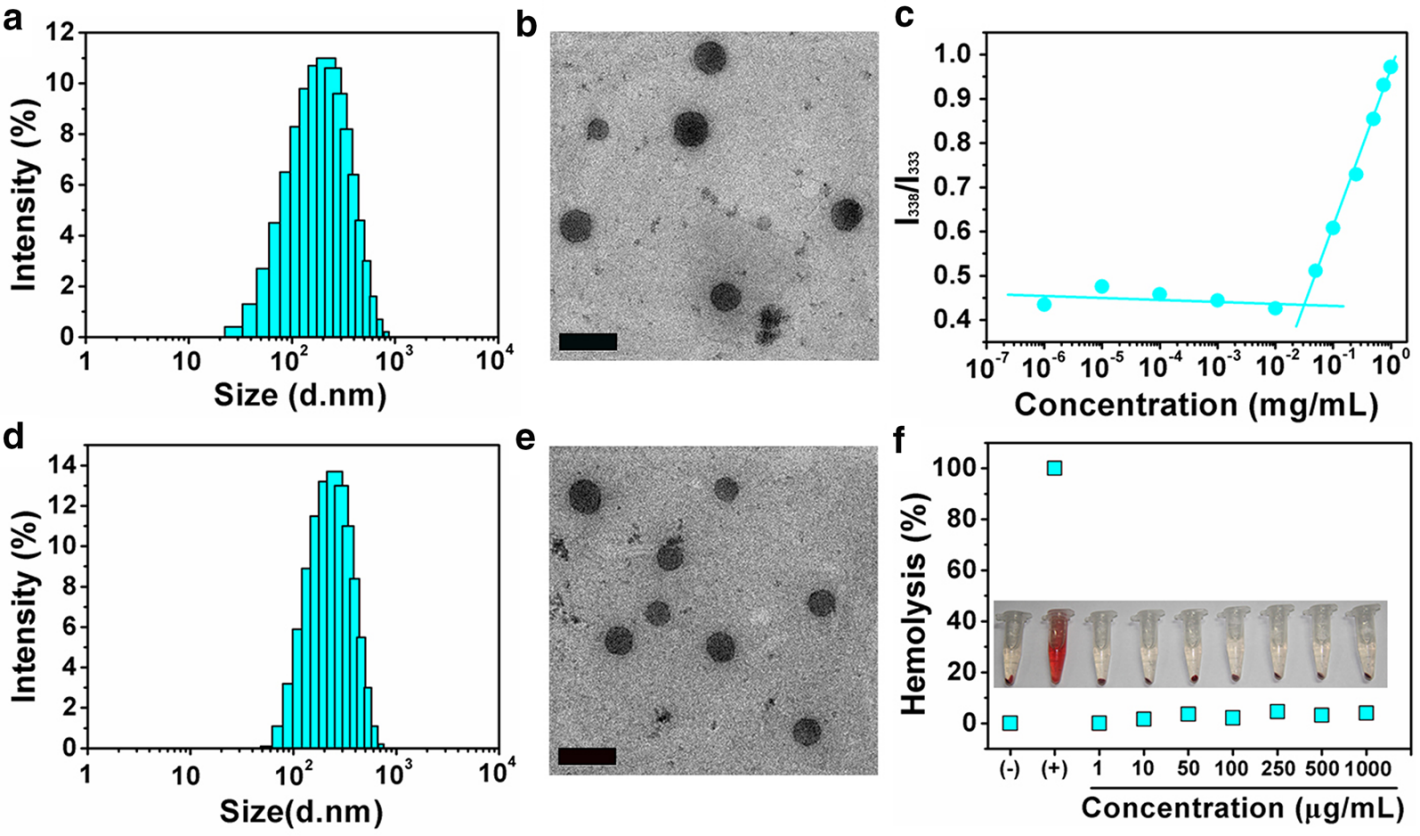
Characterization of HSC NPs and HSCI NPs. **a** Hydrodynamic diameter of HSC NPs analyzed by DLS. **b** TEM image of HSC NPs. The scale bar: 200 nm. **c** Critical aggregation concentration (CAC) curve of HSC conjugates. **d** Hydrodynamic diameter of HSCI NPs analyzed by DLS. **e** TEM image of HSCI NPs. The scale bar: 200 nm. **f** Hemolysis assay under different concentrations of HSCI NPs. PBS as negative control and water as positive control

The amphiphilic HSC conjugate was firstly synthesized and characterized (Additional file 1: Figures S1 and S2). Then, blank HSC nanoparticles (HSC NPs) could be obtained from amphiphilic HSC conjugate by self-assembling method under aqueous condition due to the hydrophobic interactions between cholesterol. As shown in Fig. 1a, DLS measurements revealed that the HSC NPs have a diameter of 181.1 ± 0.95 nm and PDI of 0.261. TEM images showed that the HSC NPs were spherical in shape and the diameter was 106.9 ± 15.83 nm (Fig. 1b). As an evaluation indicator for stability, we next measured the critical aggregation concentration (CAC). The CAC value of 0.03 mg/mL elucidated the remarkable stability of HSC NPs, which is critically important for in vitro and in vivo applications (Fig. 1c). The size of HSCI NPs measured with DLS was 184.1 ± 0.90 nm closing to that of HSC NPs (Fig. 1d). However, the PDI (0.145) of HSCI NPs was more desired and the size from TEM (96.9 ± 9.76 nm presented in Fig. 1e) was smaller than that of HSC NPs. We speculated that HSCI NPs became much tighter in order to evade the interaction with outside aqueous-soluble environment after incorporation of hydrophobic IR780. Furthermore, HSCI NPs exhibited good colloidal stability in various solutions including water, PBS (pH 6.8 and 7.4 respectively) and saline of 0.9%, in which the size and zeta potential could remain steady (Additional file 1: Figure S3). In addition, the HSCI NPs via this method obtained desired entrapment efficiency (EE, 96.6%) and high drug-loading (DL, 4.6%) of IR780. The negative surface charge of HSCI NPs also revealed the delocalization of HA backbone on the surface of the NPs. The high negative surface charge (26 ± 0.6 mV) would help maintain the stability of HSCI NPs due to electrostatic repulsion. To assess blood compatibility of HSCI NPs, hemolysis analysis experiment was then conducted. As expected, the HSCI NPs would not drive erythrocytes to release hemoglobin, which indicated the good hemocompatibility with a hemolysis rate of ≤ 4.5% (Fig. 1f). All above results indicated that our proposed HSCI NPs had the properties of uniform distribution and morphology, excellent stability and remarkable hemocompatibility, which were basic requirements for further in vivo studies.

### Programmed photoactive therapy in vitro

**Fig. 2:**
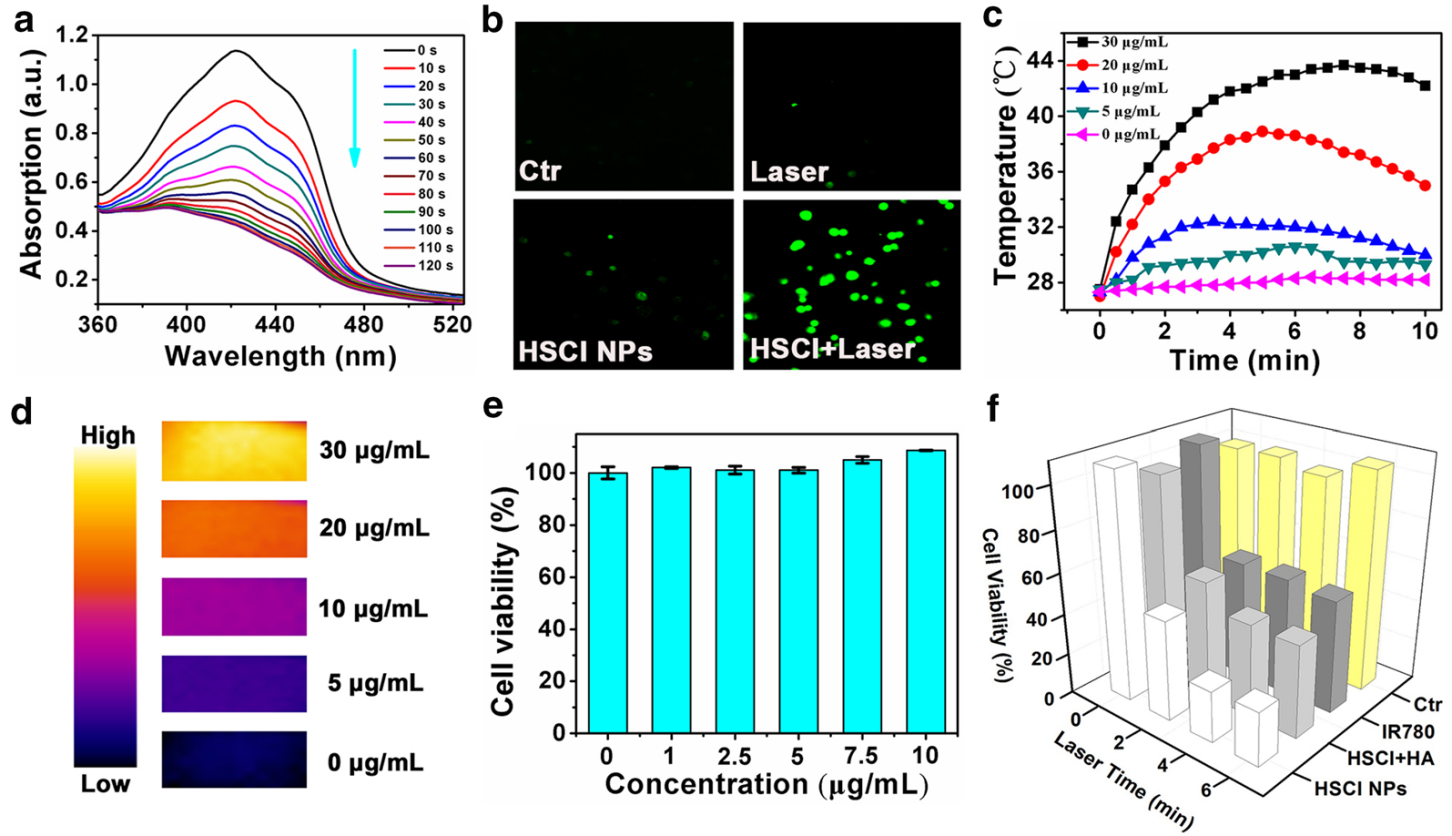
**a** The change profiles of DPBF as ROS detection probe. **b** Confocal fluorescence images for observing ROS in cells at respective treatments. **c** Temperature changing profiles as a function of time for HSCI NPs with various concentrations under laser irradiation. **d** Thermal infrared pictures of HSCI NPs implemented by NIR laser. **e** HSCI NPs toxicity assessment cell viabilities after at various concentrations with incubation time of 24 h. IR780’ concentration as reference. **f** Cell viabilities evaluation treated with HSCI NPs photo-ablation under 808 nm NIR laser for various time

Based on the good properties of HSCI NPs, the in vitro anti-cancer studies were then explored. Firstly, we used 3-diphenylisobenzofuran (DPBF) probe to detect ROS level. As shown in Fig. 2a and Additional file 1: Figure S4, HSCI NPs would generate the ROS from IR780 under the laser irradiation of 808 nm. Interestingly, we found that only the starting laser exposure (0–2 min) could induce ROS generation, while there was no ROS generation after 2 min. At the same time, although the HSCI NPs showed photothermal heating effect, the increased temperature (< 40 °C) under laser irradiation was not high enough to trigger cell death (Fig. 2c). ROS generation can be also observed by intracellular uptake and detection with relevant probe (Fig. 2b). After 2 min, the PTT would act as decisive impact for anti-cancer effects based on the fact of the fast increasement of temperature and it had reached the demand for PTT (Fig. 2c), while the HSCI NPs would not continually produce ROS (Additional file 1: Figure S4). We also found that the photothermal effect of HSCI NPs also possessed obvious concentration-dependent property (Fig. 2d, Additional file 1: Figure S5). Subsequently, we evaluated the cell cytotoxicity effect of HSCI NPs without laser treatment. As depicted in Fig. 2e, there was no obvious cytotoxicity appearing under the treatment of only HSCI NPs within 10 µg/mL (IR780 concentration as reference) (Additional file 1: Figure S6), which indicated the negligible cytotoxicity of HSCI NPs.

Next, we evaluated the cell cytotoxicity effect of HSCI NPs with different patterns of laser treatment (Fig. 2f). In the cells treated with short time of laser (2 min), the inhibition efficiency of MDA-MB-231 cells reached to 48.59%. As demonstrated before, during this time the temperature was not high enough to kill cancer cells, which demonstrated that the cell cytotoxicity was mainly due to ROS production. At subsequent laser irradiation (> 2 min), we found that the cell viability was remarkably reduced. Under the same time for laser irradiation for 6 min, the cell viability of HSCI NPs treatment (26.17%) was lower than free IR780 treatment (54.82%). According to our above discussion, as a type of small molecule, free IR780 entered cells mainly depending on the concentration gradient, which illustrated that IR780 concentration inside cells would not exceed the concentration in the medium. However, the cellular uptake way of NPs was through endocytosis-mediated pattern. As active uptake pathway, more IR780 could be delivered into cells by nanoparticles and IR780 in the cells could exhibit higher concentration, thus presenting well PTT effect compared with free IR780.

**Fig. 3:**
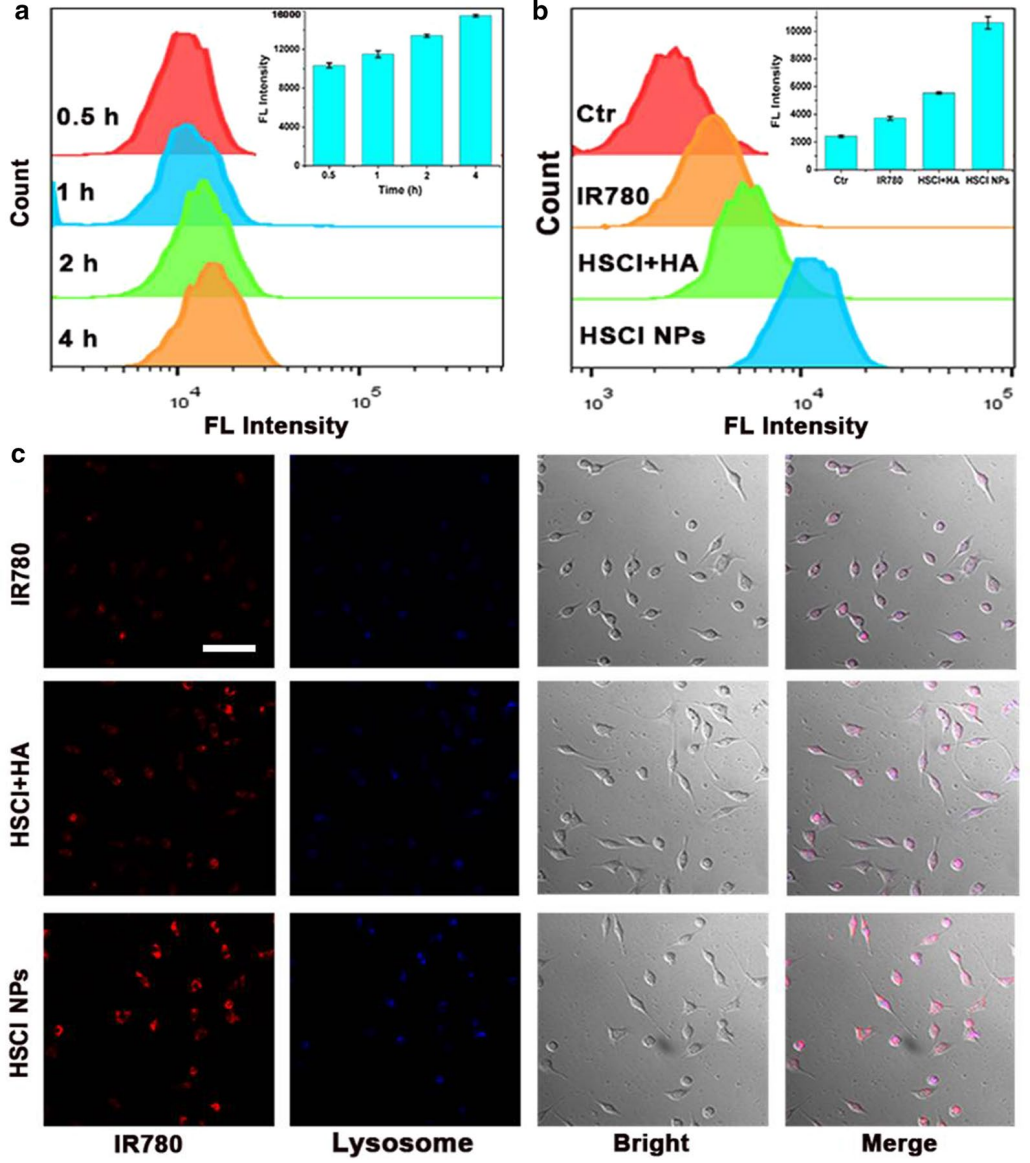
Evaluation of cellular uptake in vitro. **a** Quantized fluorescence intensity measured with flow cytometry. The cells were treated by HSCI NPs over various times. **b** Quantized fluorescence signal of cells incubated with saline, IR780, HSCI + HA and HSCI NPs respectively for 0.5 h. **c** Confocal microscopy pictures of cells at different treatments respectively with saline, free IR780, HSCI NPs + HA and HSCI NPs, the lysosome was stained with Lyso-Tracker Green. The scale bar is 100 μm

The anti-cancer effects of HSCI NPs were also evaluated by the pretreatment of MDA-MB-231 cells with free hyaluronic acid (10 mg/mL) before incubation with HSCI NPs. The results showed a remarkably decreased anti-cancer efficacy and it was still higher compared to free IR780 treatment. In addition, flow cytometry (Fig. 3a) and confocal microscopy (Fig. 3c and Additional file 1: Figure S7) assays were used to evaluate cellular uptake and intracellular distribution of HSCI NPs. The obtained data showed that the targeting ability of HSCI NPs and HSCI NPs could deliver IR780 into cancer cells more effectively than free IR780. Collectively, the above results interpreted that HSCI NPs might be applied as a targeted PPAT platform, in which PDT functioned as the first step due to the high ROS generation and PTT functioned as the second step due to the high increased temperature.

### Photoacoustic and fluorescence targeting imaging in vivo

**Fig. 4:**
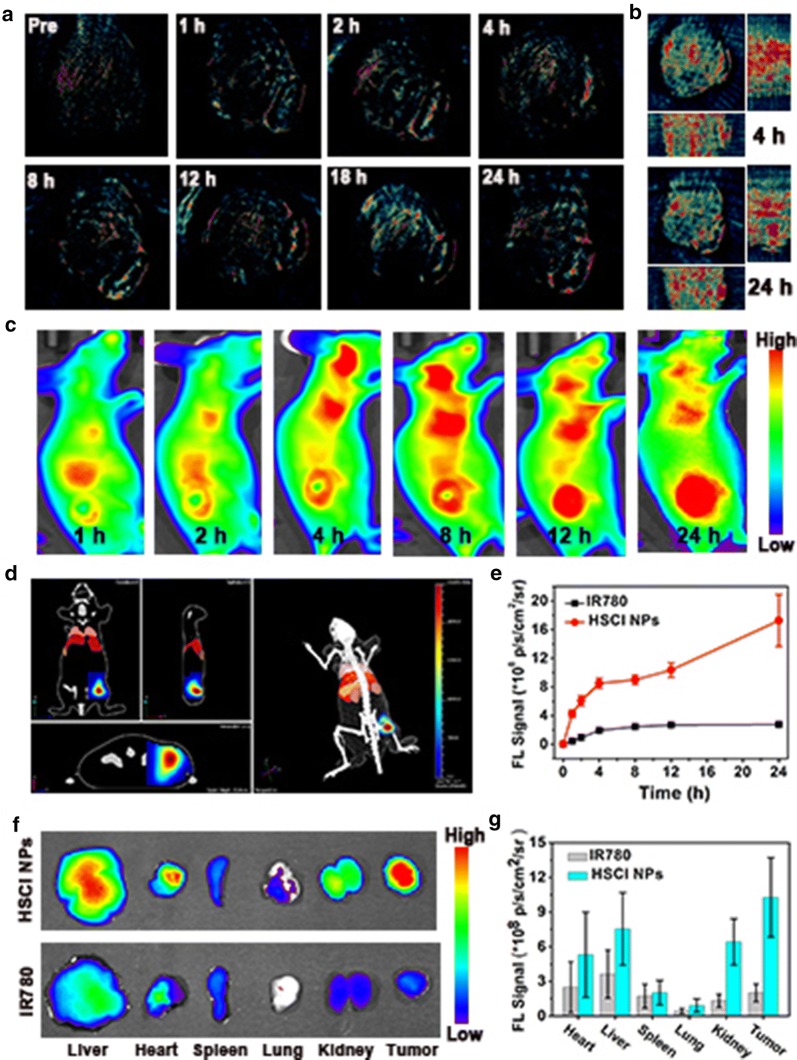
Fluorescence and MSOT imaging assessments of HSCI NPs in vivo. **a** MSOT imaging at various time-points after intravenous injection of HSCI NPs. **b** MSOT 3D imaging of tumor at 4 h and 24 h respectively. **c** Fluorescence imaging at different times of tumor via tail vein injection. **d** 3D reconstructed transillumination fluorescence imaging of HSCI NPs. **e** Quantitative fluorescence signal value of tumor at different times. **f** Fluorescence pictures of the major organs and tumor. **g** Signal quantitative statistics of major organs and tumor ex vivo

Next, we observed the accumulation behaviors of HSCI NPs with the fluorescence and MOST imaging model. The results from Additional file 1: Figure S8-A and -B revealed that the relationship between PA intensity and concentrations of HSCI NPs as well as excited wavelength. The subsequent quantitative results illustrated that the concentration of HSCI NPs ranging from 0 to 10 µg/mL was linearly dependent with PA signal value (Additional file 1: Figure S8-C). Next, MOST imaging was conducted to evaluate noninvasively accumulation behaviors of HSCI NPs in vivo. As shown in Fig. 4a and b, the PA signal in HSCI NPs-treated group presented high tendency of accumulation at tumor site compared to that of free IR780 (Additional file 1: Figure S9). And the signal gradually enhanced to maximum at 24 h post-injection (Additional file 1: Figure S10), which indicated the efficient targeting performance and MOST imaging ability of HSCI NPs. As an excellent fluorescence probe, the in vivo fluorescence imaging ability of HSCI NPs was also evaluated. Compared with MSOT imaging pattern, NIR-fluorescence imaging with high sensitivity was broadly applied as a tool to real-time monitor accumulation behaviors of nanoparticles. As shown in Fig. 4c, the fluorescence intensity was gradually enhanced at tumor sites under high sensitivity condition. It’s noteworthy that fluorescence signal can be only detected on the tumor site after injected over 24 h, which turns out the excellent tumor-targeted performance benefiting from active targeting ability of hyaluronic acid. 3D transillumination imaging was also conducted to observe depth distribution of fluorescence signal. As depicted in Fig. 4d and Additional file 2: Movie S1, the fluorescence signal can be observed at the inside of tumor turning out excellent targeting and penetration performance of HSCI NPs. The results of quantized intensity profile (Fig. 4e) from tumor site was in accordance with that of MSOT imaging. To further investigate the biodistribution of HSCI NPs, the main viscera were collected for further fluorescence imaging at the end of treatment period. As depicted in Fig. 4f and g, the HSCI NPs mostly accumulated at the tumor and liver sites. By contrast, the most fluorescence signal was predominantly detected at liver and there was little signal at tumor sites at the IR780-treated mice. Taken together, the HSCI NPs possessed excellent ability of dual-modal imaging in vivo and could accumulate effectively at tumor site by its active targeting ability, which encouraged great confidence for subsequent tumor-inhibition photoactive therapy.

### In vivo programmed photoactive therapy efficacy evaluation

**Fig. 5:**
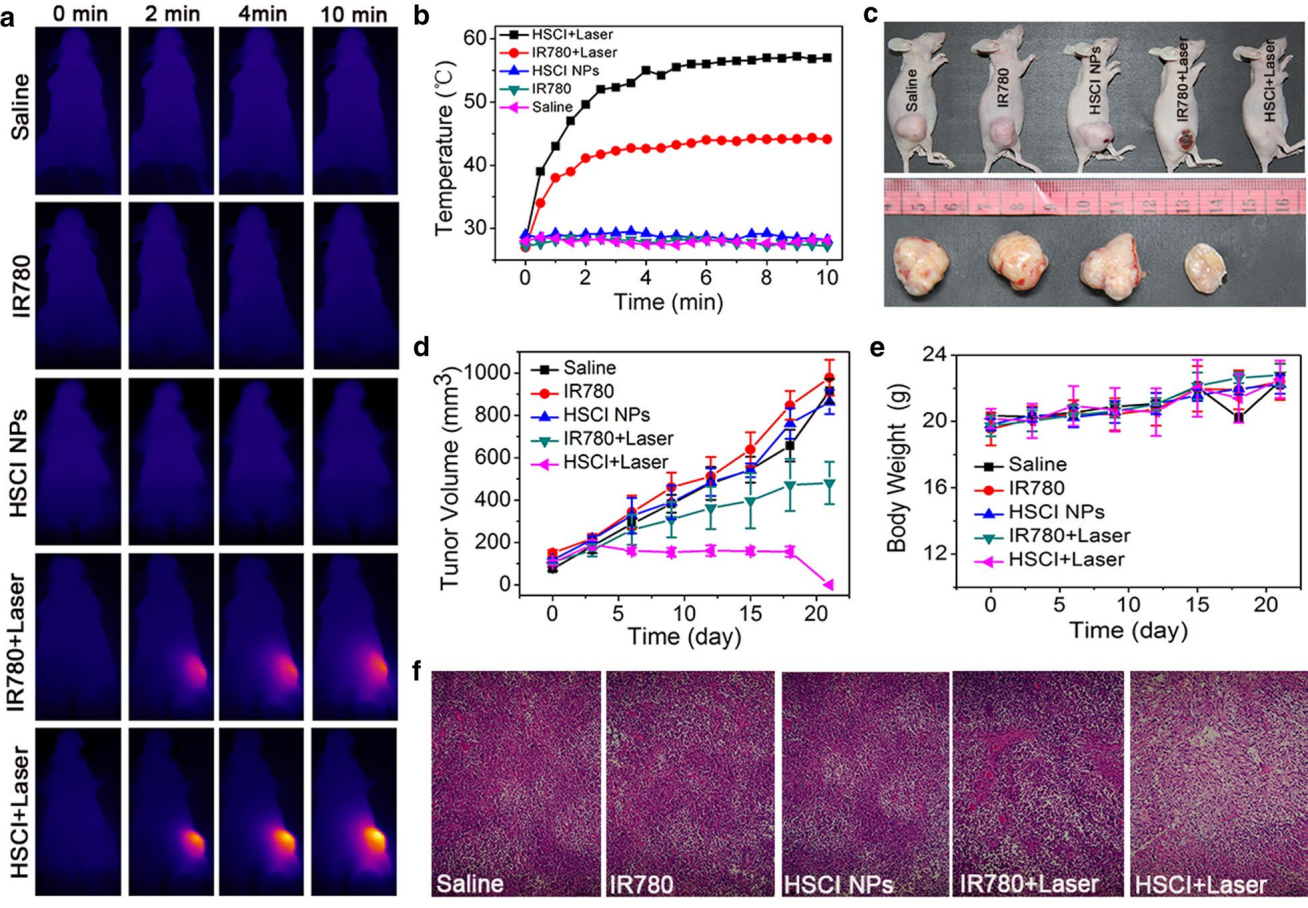
Programed photoactive therapy (PPAT) evaluation in vivo. **a** Thermal IR pictures of mice treated with saline, free IR780, HSCI, IR780 + laser and HSCI + laser. **b** Temperature profile of tumors in mice treated with different formulations. **c** Representative pictures of mice and tumors at the end of recording time. **d** Tumor volumes were measured and calculated every 3 days. **e** Body weight curves of mice treated with corresponding groups. **f** H&E staining of tumor sections

**Fig. 6:**
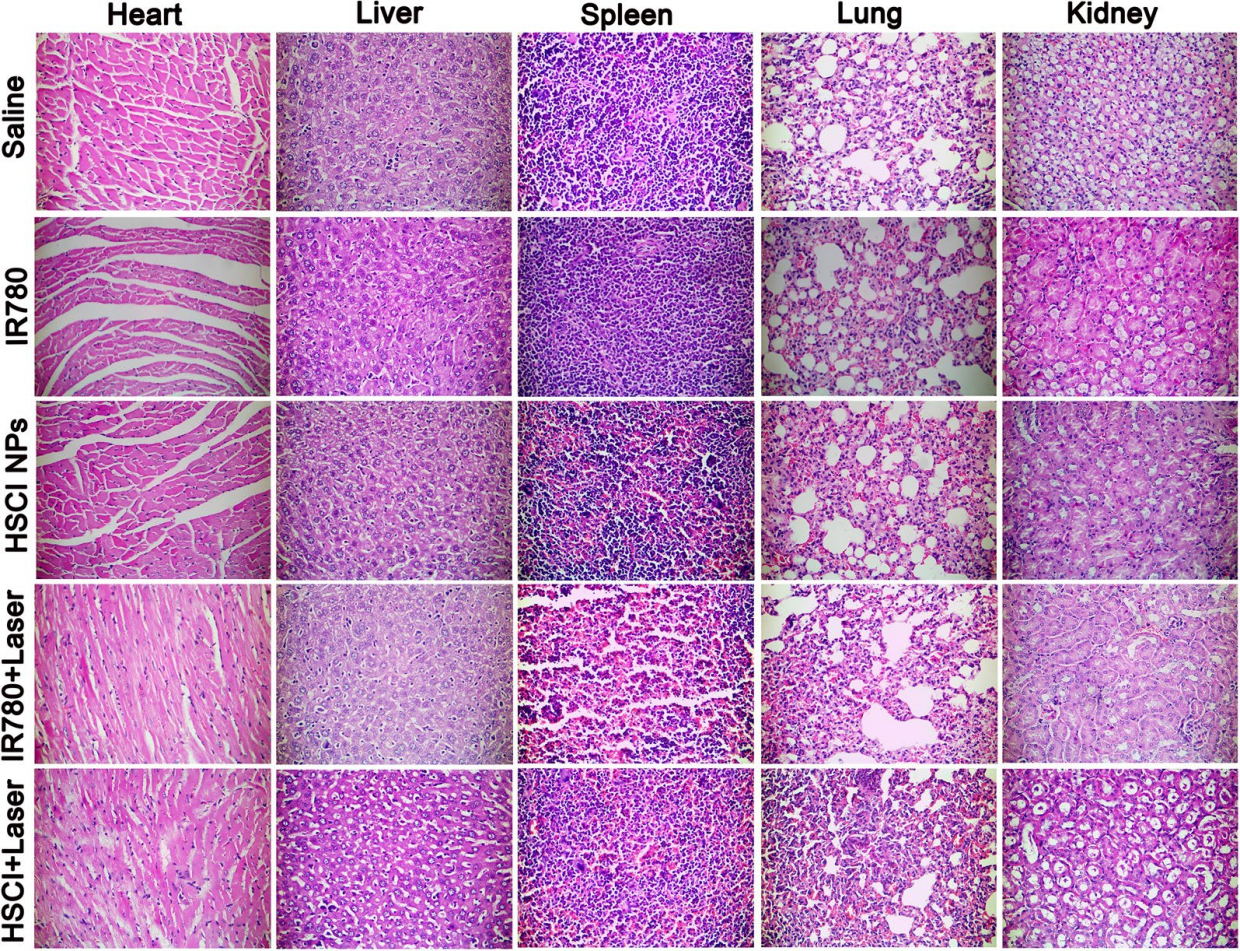
Representative H&E section of major organs collected at the end of treatment period with different groups (saline as control, free IR780, HSCI NPs, IR780 + laser and HSCI + laser)

Next, the in vivo photoactive therapy efficacy was evaluated by using established animal xenograft tumor model of triple negative breast cancer. Encouraged by the well photoactive therapy results in vitro, infrared thermal camera was firstly used to track temperature changes at tumor site (Fig. 5a, b). The group treated with HSCI NPs with laser exposure over 10 min showed more obvious increasing temperature profile and it could reach to 58.5 °C. In comparison, the group of free IR780 with laser treatment just showed sluggish temperature climbing to only 44.3 °C at the same time. As expected, HSCI NPs with laser exposure presented more obvious tumor growth-inhibition effect (Fig. 5c, d) compared with other groups. It’s worth noting that the tumor in the mice-treated with HSCI NPs would scab after laser irradiation and showed a swoop of tumor size during 18th and 21th day (Fig. 5d). In addition, the body weight showed no obvious difference at each group, indicating the bio-compatibility and safety of HSCI NPs (Fig. 5e). To further monitor the photoactive therapy efficacy, we conducted the tumors sections for H&E staining and obvious plasmorrhexis at the group of HSCI NPs with laser irradiation could be seen (Fig. 5f). As shown in Fig. 6, no obvious morphological differences were observed among all groups. All above results demonstrated the excellent phototherapy activity of HSCI NPs with desired biosafety.

## Conclusion

Taken together, we constructed “from one to all” theranostic nanoparticles by self-assembling of hyaluronic acid-cystamine-cholesterol conjugate, in which IR780 was simultaneously incorporated. The HSCI NPs could realize specific accumulation at the tumor site due to the active targeting specificity of hyaluronic acid for cancer cells over-expressing CD44 receptor. In addition, the in vivo biodistribution and accumulation behaviors could be monitored and tracked through the fluorescence and photoacoustic imaging abilities of loaded IR780. NIR laser of 808 nm with penetrating deeply tissue was conducted for programmed photoactive therapy (PPAT, PDT followed by PTT), which could inhibit tumor growth efficiently. In addition, the HSCI NPs possessed excellent biosafety. The overall data demonstrated that the constructed HSCI NPs have a great potential to be used as a tumor-targeted dual-modal imaging platform and exhibited programed photoactive therapy (PPAT) pattern with good biocompability.

## Method and materials

### Materials

Hyaluronic acid with molecular weight 10 kDa was obtained from Freda Biochem company.11-Chloro-1,1′-di-*n*-propyl-3,3,3′,3′-tetramethyl-10,12-trimethyleneindatricarbocyanine iodide (IR780) and Cholesteryl chloroformate were purchased from Alfa Aesar Co. Cystamine dihydrochloride, *N*-hydroxysuccinimide (NHS) and 1-ethyl-3 (3-dimethylaminopropyl) carbodiimide (EDC) were purchased from Sigma-Aldrich (St. Lousi, MO, USA). Glitathione (GSH) was purchased from Aladdin Reagent Database Inc. All other chemicals were analytical grade and used without further purification. Ultrapure water (deionized (DI) water) was supplied by a Milli-Q water system (Millipore, USA).

### Preparation and characterization of HA-SS-Chol (HSC) conjugates

The amphiphilic HSC conjugates were firstly synthesized according to the method reported previously with some modifications (Additional file 1: Figure S1) [37]. Cystamine modified cholesterol (Chol-SS) was synthesized by coupling cholesterol chloroformate with primary amine group of cystamine. In brief, cysteamine dihydrochloride (1.35 g, 6 mmol) was dissolved in mixed solution (180 mL, volume ratio = 10:5:4) of acetonitrile (CH_3_CN), dichloromethane (CH_2_Cl_2_) and triethylamine(TEA). After stirring for 3 h at 4 °C and another 1 h at room temperature, cholesterol chloroformate (0.27 g, 0.6 mmol) dissolved in CH_2_Cl_2_ (10 mL) was dropwise added into the mixed solution. After stirring for another 24 h, the reaction mixture was washed with DI water. The mixture was then moved to rotary evaporation following freeze-drying. Afterwards, the backbone of HA was chemically modified with the hydrophobic Chol-SS by help of EDC and NHS. HA (100 mg, 264 µmol) and Chol-SS (30 mg, 52.8 µmol) were added into H_2_O/THF (50 mL, volume ratio = 1:1) and dissolved via ultrasound about 30 min. EDC (50.424 mg, 264 µmol) and NHS (30.36 mg, 264 µmol) were dropwise added into the mixed solution. After stirring for 24 h, the reaction was dialyzed with DI water and freeze-dried gaining HA-SS-Chol conjugates. Its chemical structure was characterized by using infrared spectrometer (Spectrum One, Perkin Elmer Instruments Co. Ltd. USA).

### Critical aggregation concentration of HSC

Pyrene was used as the fluorescent probe to assess the critical aggregation concentration of HSC conjugates. The solutions ranging from 1.0 × 10^−7^ to 2.0 mg/mL were added respectively to pyrene solution (6 × 10^−7^ M). The above mixture was sonicated over 30 min and kept for 2 h to trigger HSC self-assembling into nanoparticles. Then, the fluorescent spectrum of mixture was conducted on a fluorescence spectrophotometer (F-4500, Hitachi). The fluorescent ratio of I_338_/I_333_ was calculated as a function of logarithm HSC concentration.

### Preparation and characterization of HSC-IR780 nanoparticles (HSCI NPs)

IR780-incorporated HSCI NPs were fabricated by self-assembling method [38]. Mixed solutions of HA-SS-Chol in H_2_O (2 mg/mL, 5 mL) and IR780 in CH_3_CN (1 mg/mL, 0.5 mL) was sonicated for 30 min using a probe-type ultrasonicator at 100 w/2 s on/3 s off in an ice bath. After dialyzing against distilled water overnight, the solution was filtrated through a 450 nm pore-sized microporous membrane. The entrapment efficiency (EE) and drug-loading (DL) was obtained according to the following equations:

$$ {\text{EE (\%)}} = \frac{{{\text{weight of IR78}}0{\text{ in micelles}}}}{{{\text{weight of IR78}}0{\text{ fed initially}}}} \times 100\% $$

$$ {\text{DL}}\left( {{\text{wt}}\%} \right) = \frac{{{\text{weight of IR78}}0{\text{ in micelles}}}}{{{\text{weight of IR78}}0{\text{ in micelles}} + {\text{weight of conjugates fed initially}}}} \times 100\% $$

HSC NPs were prepared as the same protocol without joining IR780 into CH_3_CN. Dynamic light scattering (DLS) measurements (Nano-ZS, Malvern instruments, UK) was used to measure hydrodynamic size and polydispersity (PDI). Measurement temperature were set value of 25 °C. Transmission electron microscopy (TEM, Tecnai G2 20 S-TWIN, Perkin Elmer Instruments Co.Ltd. USA) was conducted for further evaluation of morphology and size.

### Cell viability assays

MDA-MB-231 were incubated in DMEM media containing 10% FBS and at 37 °C in a moist atmosphere with 5% CO_2_. MDA-MB-231 cells were transfered into 96-well plates with a density of 5 × 10^3^ cells/well. After incubation overnight, 100 µL of medium containing free IR780 or HSCI NPs (10 µg/mL of IR780) were added into plates replacing the origin medium. After incubation for 4 h, the cells were washed with fresh medium twice and laser (808 nm, 0.8 W/cm^2^, 0 ~ 6 min) was conducted on cells for programed photoactive therapy. After 12 h incubation, CCK-8 assays were used to assess cell viability. For blocking assay, HA was added into the plates ahead of 2 h.

### In vitro cellular uptake evaluation

MDA-MB-231 cells (2 × 10^4^/well) were seeded on the chambered cover-glass (Lab-Tek, Nunc, USA). After incubation overnight, new medium containing IR780 (10 µg/mL), HSCI NPs (10 µg/mL of IR780 concentration) or HSCI NPs co-incubation with HA (10 mg/mL, HSCI NPs + HA) were respectively added. After incubation 30 min at 37 °C, the cells were washed thrice with PBS and stained with lysosome-green for 15 min. Then, the cells were washed thrice with PBS. Cellular uptake evaluation was conducted by confocal laser scanning microscope (Perkin Elmer, Ultra View Vox system, USA).

### Animals studies

Female BALB/c nude mice (6–8 weeks) were provided with Vital River Laboratory Animal Technology Co. Ltd. All relevant experiments were carried out following the ethical rules enacted by Experimental Animal Ethics Committee in Beijing. MDA-MB-231 cells (5 × 10^6^) were injected on the right flank of the mice to establish tumor-bearing mice. When the volume of tumors reached to 100–200 mm^3^, further studies including imaging and therapy experiments were conducted.

### Tumor-targeting imaging analysis in vivo

The mice were divided into two groups, IR780-treated mice (0.7 mg/kg of IR780) and HSCI NPs-treated mice (0.7 mg/kg of IR780). After tail vein injection, images with multi-spectral fluorescence (Cri-M2, CRI USA) and photoacoustic (M-128, iThera GER) living animal imaging system were taken at different time-points. Then major organs and tumor tissue were dissected for fluorescence imaging ex vivo by the Maestro imaging system.

### In vivo tumor-inhibition assessment

All mice were randomly separated into five groups. The mice were injected with different formulations through tail vein injection, including saline, IR780 (1.4 mg/kg), HSCI NPs (1.4 mg/kg of IR780), IR780 + Laser (1.4 mg/kg and 0.8 W/cm^2^ 10 min), HSCI NPs + Laser (1.4 kg/mg equivalent for nanoparticles and 0.8 W/cm^2^ 10 min). After the injection for 12 h, the temperature profiles of tumor during laser irradiation was recorded by thermal imager (Ti400EN, FLUKE USA). Mice body weights and tumor volume were recorded every 3 days. The duration experimental time is 3 weeks. And the formula of tumor volume is “length × width^2^/2”.

## Additional files

**Additional file 1: Figure S1.** Chemical structures and synthetic route of hyaluronic acid-cysteamine dihydrochloride-cholesterol (HSC) conjugates. **Figure S2.** FT-IR of different conjugates including cysteamine dihydrochloride-cholesterol and hyaluronic acid-cysteamine dihydrochloride-cholesterol (HSC) conjugates. **Figure S3.** Stability evaluation of HSCI NPs via sizes (A) and zeta potentials measured by DLS. **Figure S4.** UV curves of DPBF (ROS detection probe) under the laser irradiation of HSCI NPs as a function of time. **Figure S5.** Plot of temperature change as a function of different concentrations of HSCI NPs over 10 min under laser irradiation. **Figure S6.** Cell viability of different incubation groups consisting of free IR780 (A) and HSCI NPs (B). **Figure S7.** Confocal microscopy images of control group in which the cells were incubated with saline for 0.5 h, the lysosome was stained with Lyso-Tracker Green. **Figure S8.** (A) PA signal spectrum of HSCI NPs at various concentrations. (B) Linear relationship between signal intensity and concentration. (C) MSOT imaging phantoms including different concentration of HSCI NPs embedded in agar gel cylinders. **Figure S9.** MSOT imaging at different times of mice treated with free IR780. **Figure S10.** Quantification of MSOT imaging signal at tumor site at different times of mice treated with free IR780 and HSCI NPs. **Figure S11.** Fluorescence imaging at different times of mice treated with free IR780.

**Additional file 2: Movie S1.** Video of 3D reconstructed transillumination fluorescence imaging of HSCI NPs.

## Abbreviations

IR780: 2-[2-[2-Chloro-3-[(1,3-dihydro-3,3-dimethyl-1-propyl-2H-indol-2-ylidene)ethylidene]-1-cyclohexen-1-yl]ethenyl]-3,3-dimethyl-1-propylindolium iodide
HA: hyaluronic acid
HSC: hyaluronic acid-cystamine-cholesterol conjugate
HSCI NPs: nanoparticles via self-assembling of hyaluronic acid-cystamine-cholesterol conjugate and IR780
PPAT: programmed photoactive therapy
GSH: glutathione
ROS: reactive oxygen species
PDT: photodynamic therapy
PTT: photothermal therapy
MRI: magnetic resonance imaging
PET: positron emission tomography
MSOT: multispectral optoacoustic tomography
PA: photoacoustic
CAC: critical aggregation concentration

## References

[1] Li C, Zhang Y, Chen G (2017). Engineered multifunctional nanomedicine for simultaneous stereotactic chemotherapy and inhibited osteolysis in an orthotopic model of bone metastasis. Adv Mater.

[2] Lu Y, Aimetti AA, Langer R (2017). Bioresponsive materials. Nat Rev Mater.

[3] Bottini M, Rosato N, Bottini N (2011). PEG-modified carbon nanotubes in biomedicine: current status and challenges ahead. Biomacromolecules.

[4] Kuthala N, Vankayala R, Li YN (2017). Engineering novel targeted boron-10-enriched theranostic nanomedicine to combat against murine brain tumors *via* mr imaging-guided boron neutron capture therapy. Adv Mater.

[5] Ding Y, Zhou YY, Chen H (2013). The performance of thiol-terminated PEG-paclitaxel-conjugated gold nanoparticles[J]. Biomaterials.

[6] Liu Y, Ji X, Tong WWL (2018). Engineering multifunctional RNAi nanomedicine to concurrently target cancer hallmarks for combinatorial therapy[J]. Angew Chem.

[7] Sacchetti C, Motamedchaboki K, Magrini A (2013). Surface polyethylene glycol conformation influences the protein corona of polyethylene glycol-modified single-walled carbon nanotubes: potential implications on biological performance. ACS Nano.

[8] Huo S, Jin S, Ma X (2014). Ultrasmall gold nanoparticles as carriers for nucleus-based gene therapy due to size-dependent nuclear entry. ACS Nano.

[9] Zhang N, Chen H, Liu AY (2016). Gold conjugate-based liposomes with hybrid cluster bomb structure for liver cancer therapy[J]. Biomaterials.

[10] Wishart DS (2016). Emerging applications of metabolomics in drug discovery and precision medicine. Nat Rev Drug Discovery.

[11] Ho D, Wang CHK, Chow EKH (2015). Nanodiamonds: the intersection of nanotechnology, drug development, and personalized medicine. Sci Adv.

[12] Wang Y, Liu J, Ma X (2018). Nanomaterial-assisted sensitization of oncotherapy. Nano Res.

[13] Huo S, Ma H, Huang K (2012). Superior penetration and retention behavior of 50 nm gold nanoparticles in tumors. Can Res.

[14] Yang D, Yang F, Hu J (2009). Hydrophilic multi-walled carbon nanotubes decorated with magnetite nanoparticles as lymphatic targeted drug delivery vehicles. Chem Commun.

[15] Janib SM, Moses AS, MacKay JA (2010). Imaging and drug delivery using theranostic nanoparticles. Adv Drug Deliv Rev.

[16] Schick I, Lorenz S, Gehrig D (2014). Multifunctional two-photon active silica-coated Au@ MnO Janus particles for selective dual functionalization and imaging. J Am Chem Soc.

[17] Lim EK, Kim T, Paik S (2014). Nanomaterials for theranostics: recent advances and future challenges. Chem Rev.

[18] Sanhai WR, Sakamoto JH, Canady R (2008). Seven challenges for nanomedicine. Nat Nanotechnol.

[19] Wicki A, Witzigmann D, Balasubramanian V (2015). Nanomedicine in cancer therapy: challenges, opportunities, and clinical applications. J Control Release.

[20] He S, Johnson NJJ, Nguyen Huu VA (2017). Simultaneous enhancement of photoluminescence, MRI relaxivity, and CT contrast by tuning the interfacial layer of lanthanide heteroepitaxial nanoparticles. Nano Lett.

[21] Keliher EJ, Ye YX, Wojtkiewicz GR (2017). Polyglucose nanoparticles with renal elimination and macrophage avidity facilitate PET imaging in ischaemic heart disease. Nat Commun.

[22] Kim T, Lee N, Arifin DR (2017). *In vivo* micro-CT imaging of human mesenchymal stem cells labeled with gold-poly-l-lysine nanocomplexes. Adv Func Mater.

[23] Laissue PP, Alghamdi RA, Tomancak P (2017). Assessing phototoxicity in live fluorescence imaging. Nat Methods.

[24] Frangioni JV (2003). *In vivo* near-infrared fluorescence imaging. Curr Opin Chem Biol.

[25] Jokerst JV, Gambhir SS (2011). Molecular imaging with theranostic nanoparticles. Acc Chem Res.

[26] Tomography P (2012). *In vivo* imaging from organelles to organs wang, Lihong V.; Hu, Song. Science.

[27] Pu K, Shuhendler AJ, Jokerst JV (2014). Semiconducting polymer nanoparticles as photoacoustic molecular imaging probes in living mice. Nat Nanotechnol.

[28] Li K, Liu B (2014). Polymer-encapsulated organic nanoparticles for fluorescence and photoacoustic imaging. Chem Soc Rev.

[29] Kumar A, Kumar S, Rhim WK (2014). Oxidative nanopeeling chemistry-based synthesis and photodynamic and photothermal therapeutic applications of plasmonic core-petal nanostructures. J Am Chem Soc.

[30] Qian C, Feng P, Yu J (2017). Anaerobe-inspired anticancer nanovesicles. Angew Chem Int Ed.

[31] Liu Y, Liu Y, Bu W (2015). Hypoxia induced by upconversion-based photodynamic therapy: towards highly effective synergistic bioreductive therapy in tumors. Angew Chem Int Ed.

[32] Dou R, Du Z, Bao T (2016). The polyvinylpyrrolidone functionalized rGO/Bi 2 S 3 nanocomposite as a near-infrared light-responsive nanovehicle for chemo-photothermal therapy of cancer. Nanoscale..

[33] Abbas M, Zou Q, Li S (2017). Self-assembled peptide-and protein-based nanomaterials for antitumor photodynamic and photothermal therapy. Adv Mater.

[34] Jung HS, Verwilst P, Sharma A (2018). Organic molecule-based photothermal agents: an expanding photothermal therapy universe. Chem Soc Rev.

[35] Yang T, Liu L, Deng Y (2017). Ultrastable near-infrared conjugated-polymer nanoparticles for dually photoactive tumor inhibition. Adv Mater.

[36] Kim JY, Choi WI, Kim M (2013). Tumor-targeting nanogel that can function independently for both photodynamic and photothermal therapy and its synergy from the procedure of PDT followed by PTT. J Control Release.

[37] Choi K, Jang M, Kim JH (2014). Tumor-specific delivery of siRNA using supramolecular assembly of hyaluronic acid nanoparticles and 2b RNA-binding protein/siRNA complexes. Biomaterials.

[38] Yue C, Liu P, Zheng M (2013). IR-780 dye loaded tumor targeting theranostic nanoparticles for NIR imaging and photothermal therapy. Biomaterials.

