# Supplementary material for: From one to all: self-assembled theranostic nanoparticles for tumor-targeted imaging and programmed photoactive therapy
Source: J Nanobiotechnology. 2019 Feb 2;17:23. doi: 10.1186/s12951-019-0450-x (PMC6359812; doi:10.1186/s12951-019-0450-x)
Supplement: Supplementary file 1 — Additional file 1: Figure S1. Chemical structures and synthetic route of hyaluronic acid-cysteamine dihydrochloride-cholesterol (HSC) conjugates. Figure S2. FT-IR of different conjugates including cysteamine dihydrochloride-cholesterol and hyaluronic acid-cysteamine dihydrochloride-cholesterol (HSC) conjugates. Figure S3. Stability evaluation of HSCI NPs via sizes (A) and zeta potentials measured by DLS. Figure S4. UV curves of DPBF (ROS detection probe) under the laser irradiation of HSCI NPs as a function of time. Figure S5. Plot of temperature change as a function of different concentrations of HSCI NPs over 10 min under laser irradiation. Figure S6. Cell viability of different incubation groups consisting of free IR780 (A) and HSCI NPs (B). Figure S7. Confocal microscopy images of control group in which the cells were incubated with saline for 0.5 h, the lysosome was stained with Lyso-Tracker Green. Figure S8. (A) PA signal spectrum of HSCI NPs at various concentrations. (B) Linear relationship between signal intensity and concentration. (C) MSOT imaging phantoms including different concentration of HSCI NPs embedded in agar gel cylinders. Figure S9. MSOT imaging at different times of mice treated with free IR780. Figure S10. Quantification of MSOT imaging signal at tumor site at different times of mice treated with free IR780 and HSCI NPs. Figure S11. Fluorescence imaging at different times of mice treated with free IR780. [file 12951_2019_450_MOESM1_ESM.docx]

From One to All: Self-Assembled Theranostic Nanoparticles for Tumor-Targeted Imaging and Programmed Photoactive Therapy.

Xianlei Li,^a,b,^† Xuan Wang,^a,b,^† Caiyan Zhao,^a,b^ Leihou Shao,^a,b^ Jianqing Lu,^a^ Yujia Tong,^a^  Long Chen,^a,b^ Xinyue Cui,^a^  Huiling Sun,^a^  Junxing Liu,^c^  Mingjun Li,^c, *^ Xiongwei Deng,^a*^ Yan Wu^a,b,*^

a CAS Key Laboratory for Biomedical Effects of Nanomaterials and Nanosafety, CAS Center for Excellence in Nanoscience, National Center for Nanoscience and Technology, No. 11 First North Road, Zhongguancun, Beijing 100190, China.

b University of Chinese Academy of Sciences, Beijing 100049, P. R. China.

c The First Affiliated Hospital of Jiamusi University, Jiamusi 154003, China.

**
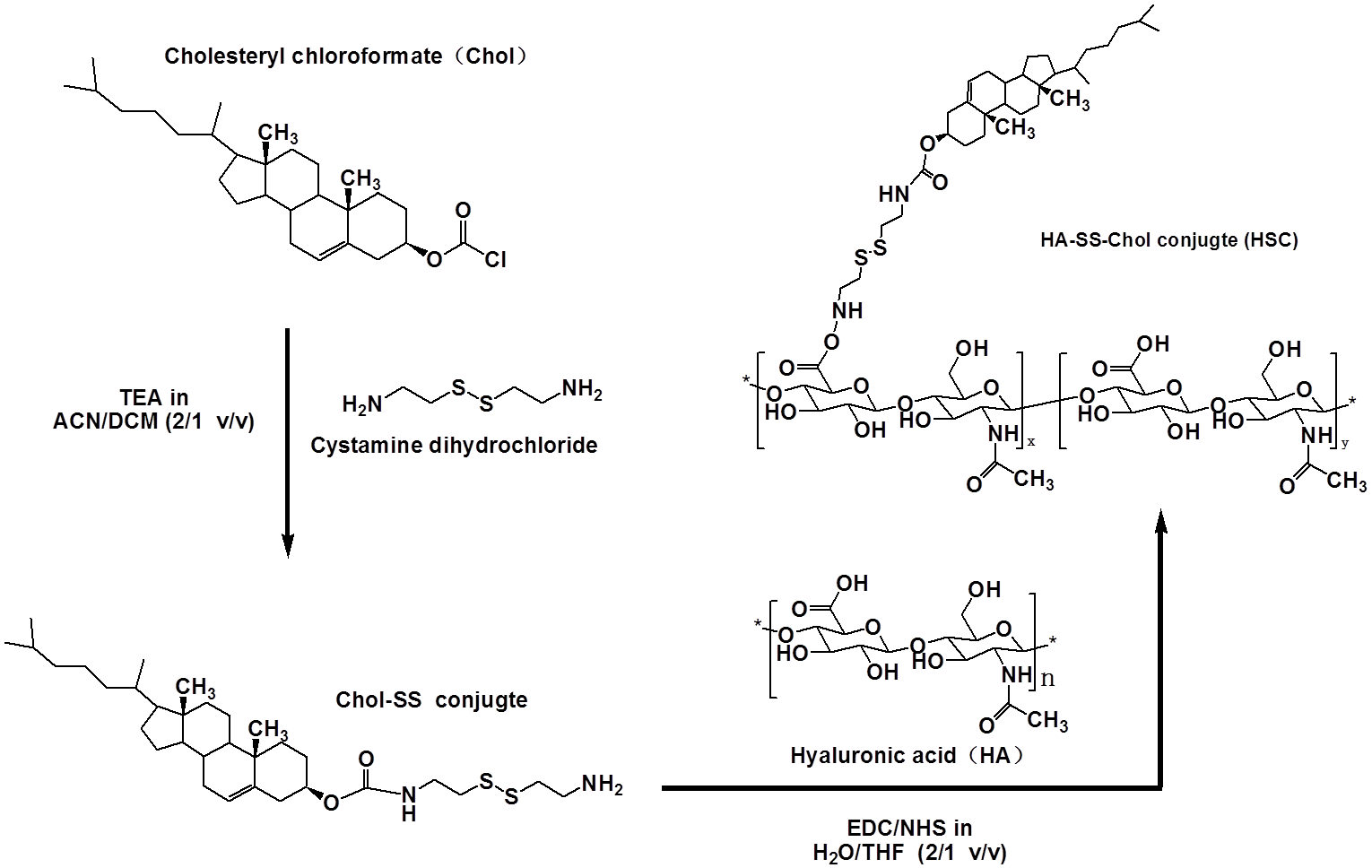
**

**Figure S1.** Chemical structures and synthetic route of hyaluronic acid-cysteamine dihydrochloride-cholesterol (HSC) conjugates.

**
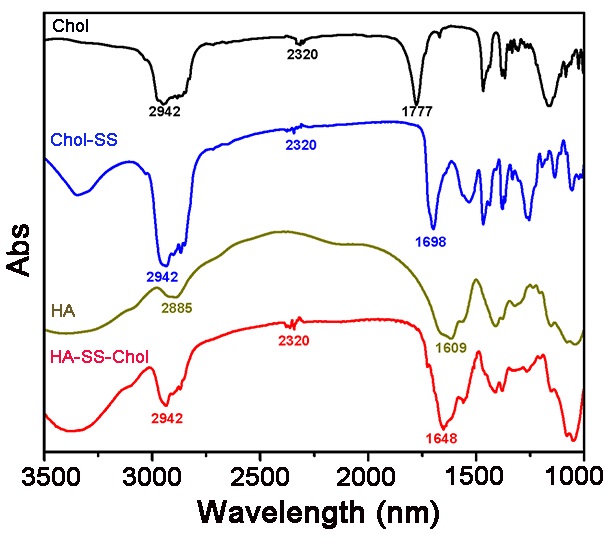
**

**Figure S2.** FT-IR of different conjugates including cysteamine dihydrochloride-cholesterol and hyaluronic acid-cysteamine dihydrochloride-cholesterol (HSC) conjugates.

**
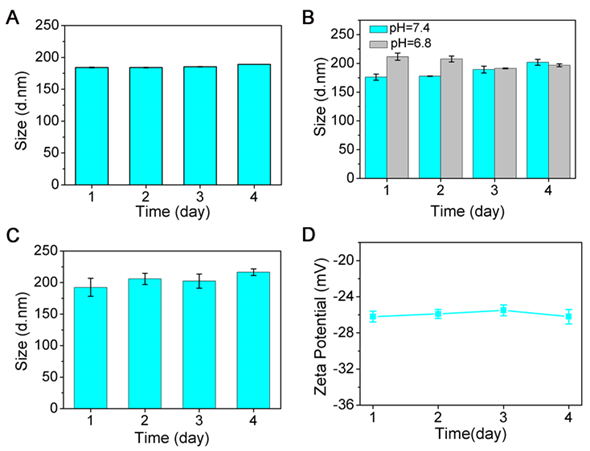
**

**Figure S3.** Stability evaluation of HSCI NPs *via* sizes under (A) H_2_O, (B) PBS at different pH (7.4 and 6.8), (C) saline of 0.9% and (D) zeta potentials measured by DLS

**
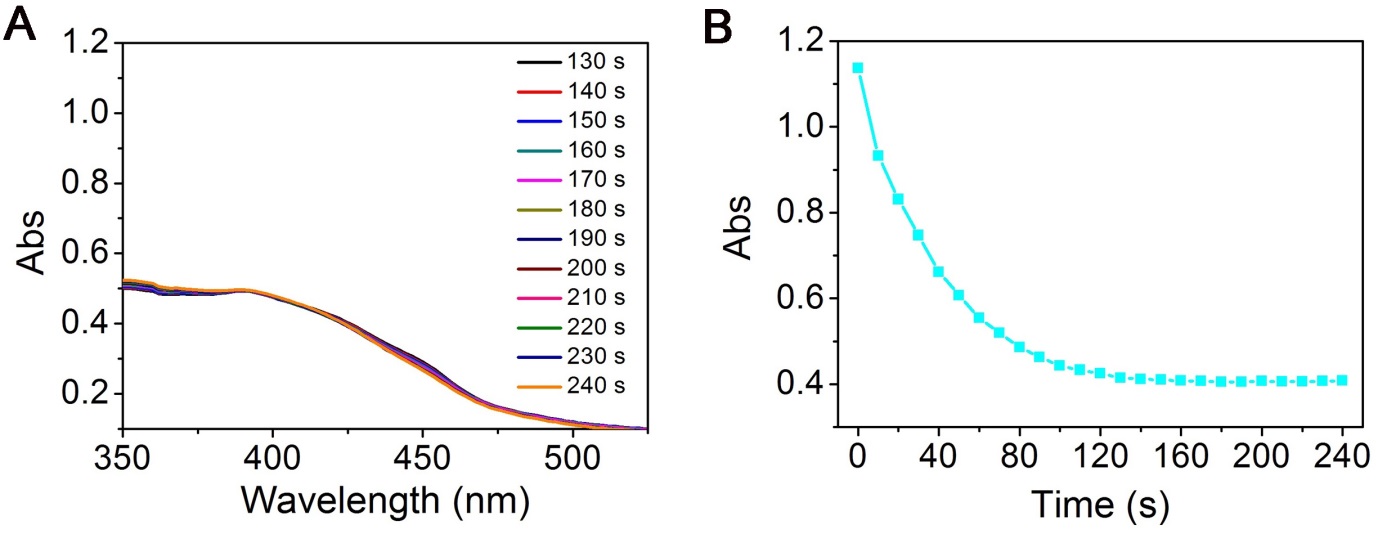
**

**Figure S4.** UV curves of DPBF (ROS detection probe) under the laser irradiation of HSCI NPs as a function of time.

**
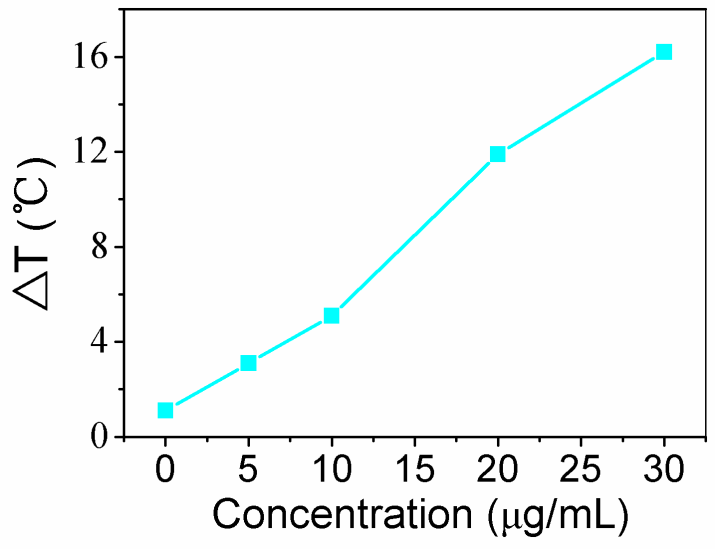
**

**Figure S5.** Plot of temperature change as a function of different concentrations of HSCI NPs over 10 minutes under laser irradiation

**
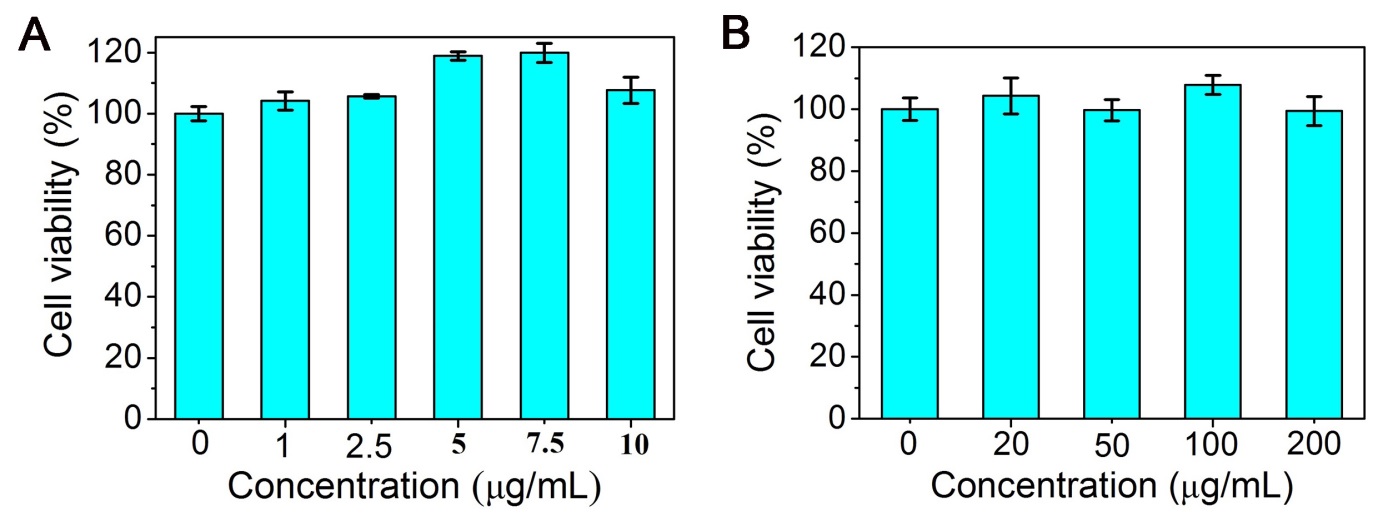
Figure S6**. Cell viability of different incubation groups consisting of free IR780 (A) and HSCI NPs (B).


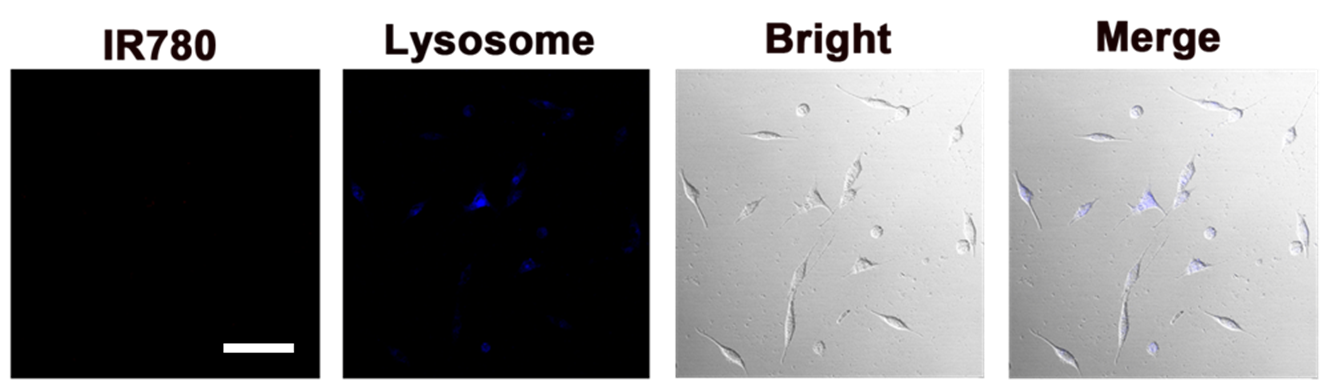


**Figure S7**. Confocal microscopy images of control group in which the cells were incubated with saline for 0.5 h, the lysosome was stained with Lyso-Tracker Green. The scale bar is 100 μm

**
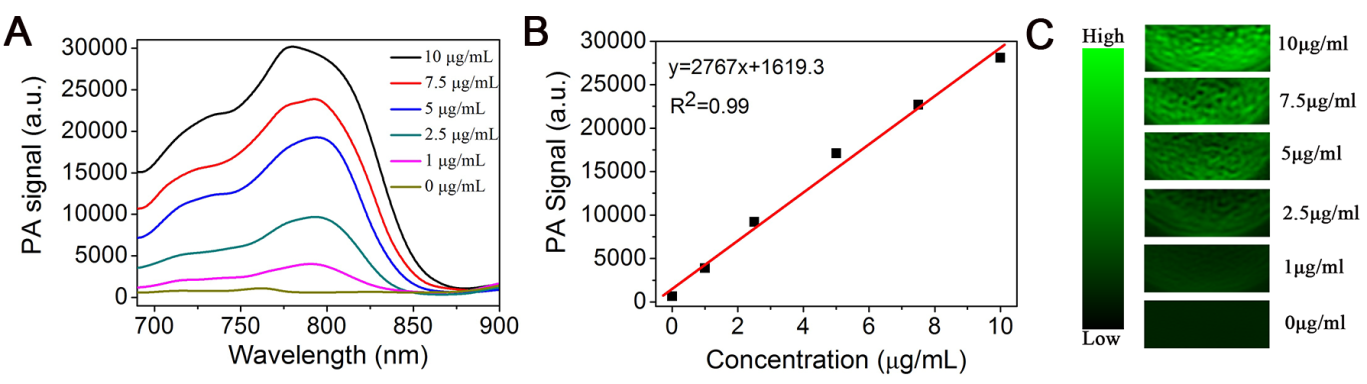
**

**Figure S8.** (A) PA signal spectrum of HSCI NPs at various concentrations. (B) Linear relationship between signal intensity and concentration. (C) MSOT imaging phantoms including different concentration of HSCI NPs embedded in agar gel cylinders.


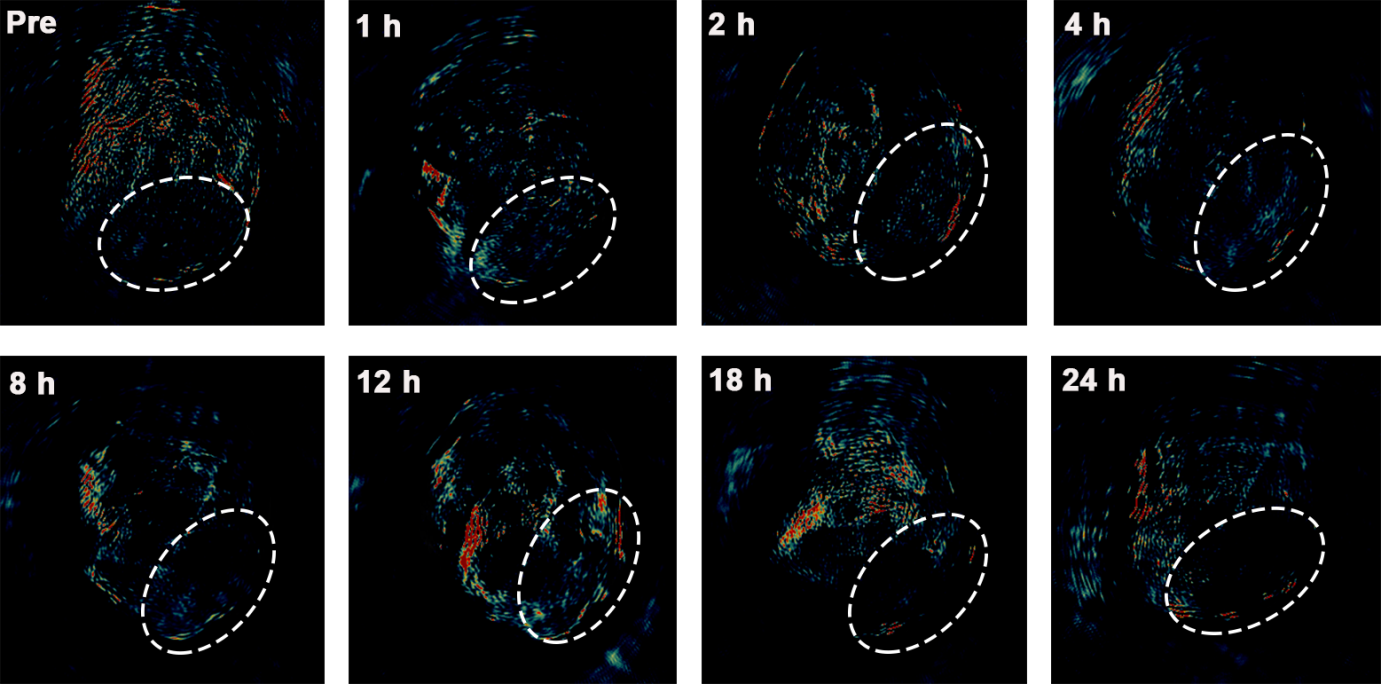


**Figure S9.** MSOT imaging at different times of mice treated with free IR780.


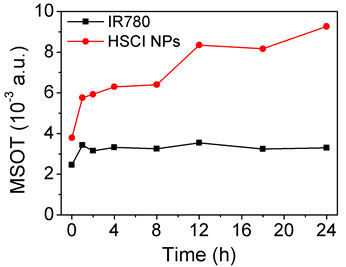


**Figure S10**. Quantification of MSOT imaging signal at tumor site at different times of mice treated with free IR780 and HSCI NPs.


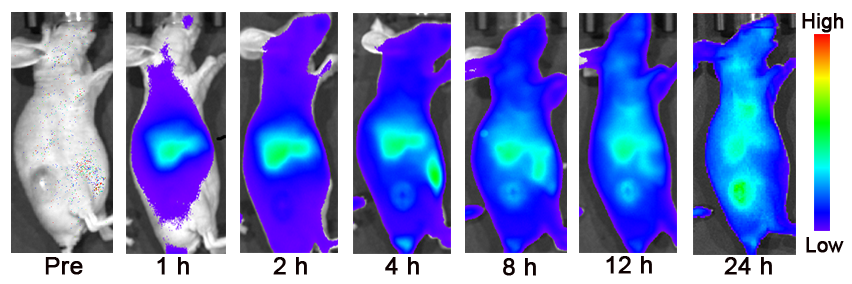


**Figure S11.** Fluorescence imaging at different times of mice treated with free IR780.
